# Advancing the Utility of DNA Origami Technique through Enhanced Stability of DNA-Origami-Based Assemblies

**DOI:** 10.1021/acs.bioconjchem.2c00311

**Published:** 2022-08-19

**Authors:** Sesha Manuguri, Minh-Kha Nguyen, Jacky Loo, Ashwin Karthick Natarajan, Anton Kuzyk

**Affiliations:** † Department of Neuroscience and Biomedical Engineering, School of Science, Aalto University, FI-00076 Aalto, Finland; ‡ Faculty of Chemical Engineering, Ho Chi Minh City University of Technology (HCMUT), 268 Ly Thuong Kiet St., Dist. 10, Ho Chi Minh City 70000, Vietnam; § Vietnam National University Ho Chi Minh City, Linh Trung Ward, Thu Duc Dist., Ho Chi Minh City 756100, Vietnam

## Abstract

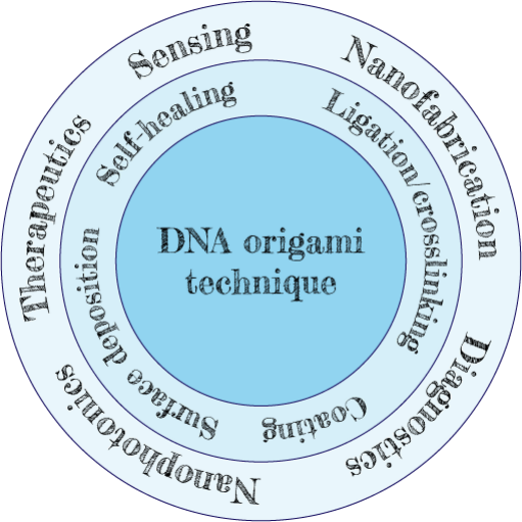

Since its discovery in 2006, the DNA origami technique has revolutionized bottom-up nanofabrication. This technique is simple yet versatile and enables the fabrication of nanostructures of almost arbitrary shapes. Furthermore, due to their intrinsic addressability, DNA origami structures can serve as templates for the arrangement of various nanoscale components (small molecules, proteins, nanoparticles, etc.) with controlled stoichiometry and nanometer-scale precision, which is often beyond the reach of other nanofabrication techniques. Despite the multiple benefits of the DNA origami technique, its applicability is often restricted by the limited stability in application-specific conditions. This Review provides an overview of the strategies that have been developed to improve the stability of DNA-origami-based assemblies for potential biomedical, nanofabrication, and other applications.

## Introduction

The use of DNA as a building block has brought a transformative change in the ability to build, organize, and modify materials.^1−3^ The inherent simplicity and programmability of the sequence-dependent base-pairing, combined with the intrinsic responsiveness of DNA to a wide range of environmental conditions (pH value, temperature, ionic strength) and external stimuli (chemical fuels, electric fields, light), makes DNA an excellent construction material for molecular engineers.^4−8^ The DNA origami (DO) technique has been proven to be particularly useful for building sophisticated nanostructures and arranging nanoparticles, fluorophores, small molecules, and proteins into complex nanoscale architectures with novel functionalities.^9−17^ In addition to using individual DO constructs as programmable assembly templates, significant efforts have been devoted to extending the DO technique to larger scales. These include DO-based structures with molecular weight up to gigadaltons and controlled organization of DO structures on surfaces.^18−27^ Furthermore, dynamic DO structures with reconfigurable structural features were built in both 2D and 3D.^28−34^ The DO technique holds enormous potential for real-life applications across diverse fields;^16,35−42^ however, challenges remain. For example, the use of DO in solution-based conditions in biomedical applications, e.g., sensing and drug delivery, is often hampered by low DO stability under nonoptimal buffer conditions and susceptibility of DO to nuclease degradation. For applications utilizing surface immobilized DO structures, preservation of nanoscale morphological features of DO constructure is typically required.^43,44^ It became imperative to gain a comprehensive understanding of the role of the surrounding environment in the structural integrity and stability of DO structures. Here, we present an overview of various strategies to improve the resilience of DO structures in application-specific conditions. In particular, we highlight several promising directions, along which the DO technique may contribute to overcoming the present technological challenges.

### Factors Influencing the Stability of DNA Origami

DNA can withstand a wide range of environments, and genetic information can be preserved over centuries.^45,46^ However, when assembled into a DO nanostructure, the structural stability is highly influenced by pH value, ionic strength, and temperature.^47^ Besides intrinsic properties of DNA, stability characteristics of DO-based constructs are also influenced by the strand routing design and structural features of DO structures. DO technique relies on two types of DNA strands, a long single-stranded DNA (ssDNA) (“scaffold”) and multiple short ssDNA strands (“staples”). DO structures are essentially composed of double-stranded DNA (dsDNA) formed through scaffold-staples hybridization and interlinked by DNA crossovers. The presence of multiple staple strands results in a large number of DNA nicks within the DO structure. Furthermore, the densely packed negatively charged DNA in a DO structure leads to strong electrostatic repulsion, which is usually screened by the presence of divalent cations (typically Mg^2+^ in mM range).

**Figure 1:**
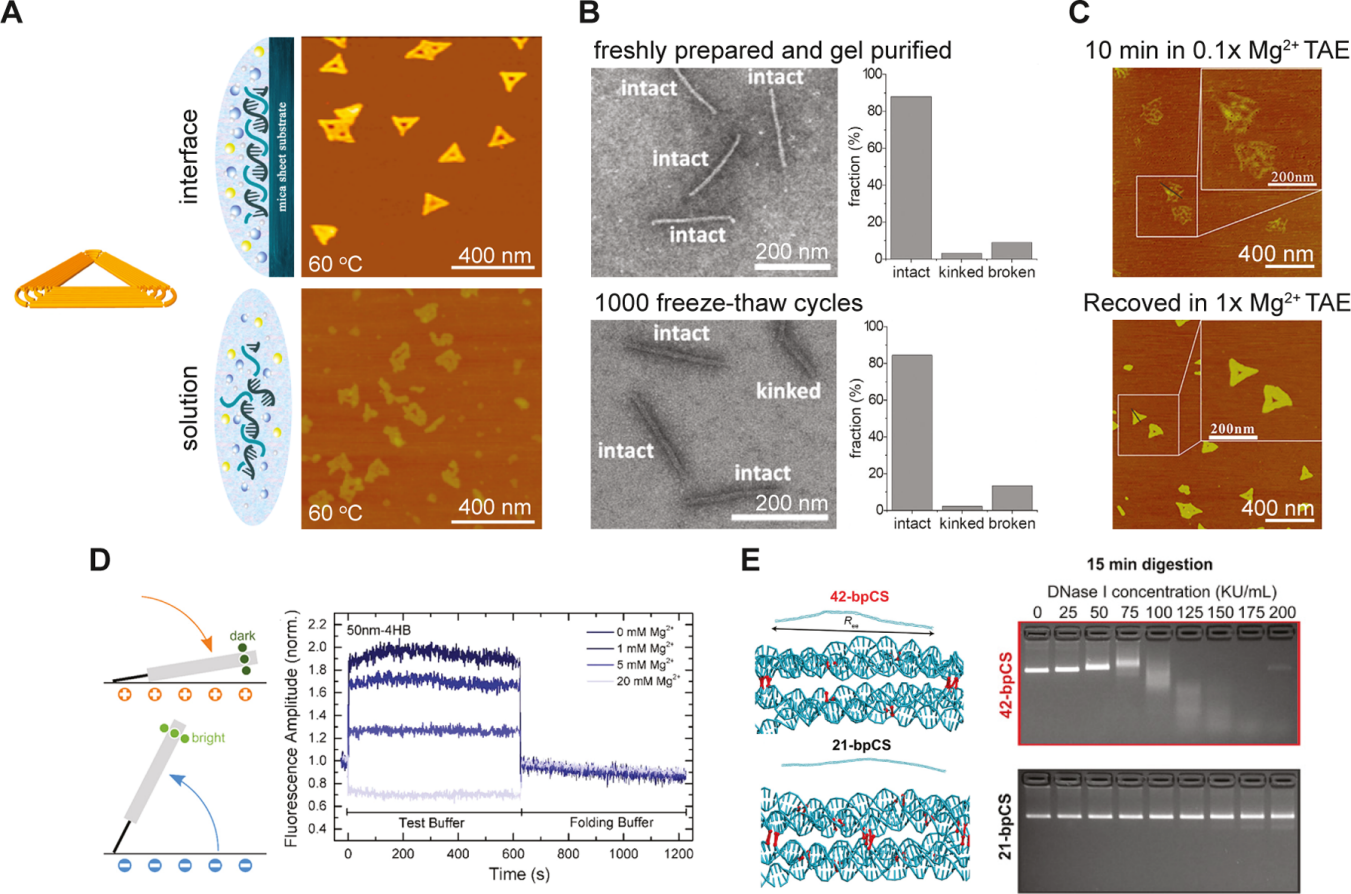
(A) DO structures exhibit higher thermal stability on an interface than in solution. AFM images of DO show the triangular origami on the mica substrates remains intact after heating to 60 °C, whereas in solution the DO structures are significantly damaged. (Adapted with permission from ref (48), Copyright 2018 American Chemical Society). (B) DO structures can withstand over 1000 freeze–thaw cycles in liquid nitrogen in the presence of glycerol. TEM images of 14-HB DO and analysis of corresponding fractions of intact, kinked, and broken DO nanostructures after assembly of agarose gel electrophoresis purification and after 1000 freeze–thaw cycles. (Adapted with permission from ref (51), Copyright 2020 John Wiley & Sons, Inc.). (C) Triangular DO structures can recover their shape after short incubation in low Mg^2+^ buffers. AFM images of partially disassembled DO after 10 min incubation in 0.1× Mg^2+^ TAE buffer and recovered structures after incubation in original 1× Mg^2+^ TAE buffer. (Adapted with permission from ref (54), Copyright 2017 IOP Publishing). (D) DO structures remain functional for electrical actuation on a surface at low Mg^2+^ concentrations. Fluorescence switching amplitudes of 50 nm long 4-HB DO at different Mg^2+^ concentrations from 0 to 20 mM. (Adapted with permission from ref (56), Copyright 2019 American Chemical Society). (E) Increased number of crossovers, i.e., at every 21-basepair (bp) interval, improves the nuclease resistance of DO structures. (Left) Molecular dynamics simulations of 6-HB DO with crossovers (highlighted in red) at 42- and 21-bp intervals. (Right) Gel electrophoresis demonstrating enhanced stability of DOs with 21-bp crossover spacing to degradation by DNase I. (Adapted with permission from ref (70). Copyright 2022 John Wiley & Sons, Inc.).

Temperature is a parameter with apparent relevance in the context of DO stability and utility for specific applications. In solution, DO structures typically suffer significant damage at elevated temperatures (Figure 1A).^48^ Depending on the DO design and buffer conditions, disassembly becomes apparent in the temperature range between 60 and 80 °C. DO stability can be drastically improved by simple deposition on a surface, e.g., DO structures adhered to mica.^48,49^ This can be explained by the restricted movement of DNA strands within DO constructs imposed by the attachment to the surface and by the decreased diffusion of single short DNA strands separated from the origami upon heating.^48^ As will be discussed later, the stability of DO structures at elevated temperatures can also be greatly improved by ligation and various cross-linking and coating approaches. Under low temperatures (−20 °C), both DNA staple strands^50^ and DO structures can remain stable over years.^51^ Remarkably, DO structures can survive over 1000 freeze–thaw cycles in liquid nitrogen when supplemented with cryoprotectants such as glycerol or trehalose (Figure 1B).^51^ This bodes well for large-scale industrial fabrication of DO structures when they will be employed in real-life applications.

In the past, it was generally accepted that a relatively high concentration of Mg^2+^ (5–20 mM range) was required for the stability of DO in solution. One possible reason for this perception is that 5–20 mM concentrations of Mg^2+^ are typically needed for high yields of DO assembly by thermal annealing.^52^ Early works on Mg^2+^-dependent DO stability suggested that unmodified origami structures are generally not stable at Mg^2+^ concentrations below 1 mM,^53^ although DO constructs partially disassembled after short incubation in low Mg^2+^ buffer and can be recovered by increasing Mg^2+^ concentration (Figure 1C).^54^ More recent works demonstrated that various DO constructs, e.g., triangle, 6-helix bundle (6-HB), and 24-HB nanostructures, are stable at low (down to low-micromolar range) Mg^2+^ concentrations,^55^ and application-specific functional features of DOs, e.g., electric field induced actuation, can also be preserved (Figure 1D).^56^ It should be noted that, in principle, Mg^2+^ is not required for assembly or storage of DO structures, and it can be substituted by monovalent cations, although usually at much higher concentrations.^57,58^ In addition, DO constructs with wireframe design^59−64^ are generally stable in buffers of lower ionic strength than origami with densely packed DNA routing. Several recent studies explored effects of high, i.e., over 100 mM, Mg^2+^ concentration on structural integrity of DO assemblies. Li and co-workers utilized all-atom molecular dynamics simulations to study ionic conductivity through DO plates.^65^ Their results indicate that DO plates adopt more compact configuration at elevated Mg^2+^ levels, which leads to reduced conductivity. Hübner et al. reported that high Mg^2+^ concentrations can induce significant structural changes to the so-called single layer DO structures.^66^ In their study, the structures adopted flat rectangular configurations at 12 nM Mg^2+^ but rolled up at high Mg^2+^ concentrations. Importantly, the structural transitions were reversible.

In terms of pH value, DO structures are generally stable in the range between 5.5 and 9.5. Although depurination of a DNA strand and hydrogen bond breaking between two hybridized DNA in the DO occurs at low (pH < 5) and high (pH > 10) pH values, fluctuation in pH is generally regarded as a less problematic issue, since a buffered environment is generally used in various applications. Nevertheless, surface deposition^67^ and surface coating^68^ were shown to increase the pH-dependent DO stability range even further.

Another common concern, especially in the context of biomedical applications, is the degradation of DO structures by nucleases.^53,69^ In addition to various coating approaches (discussed below), resilience to nucleases can be improved by incorporating more dense DNA crossovers at the design stage. It has been shown that decreasing crossover separation from 42- to 21-basepair (bp) can significantly increase DO resistance to nuclease degradation (Figure 1E).^70^

**Figure 2:**
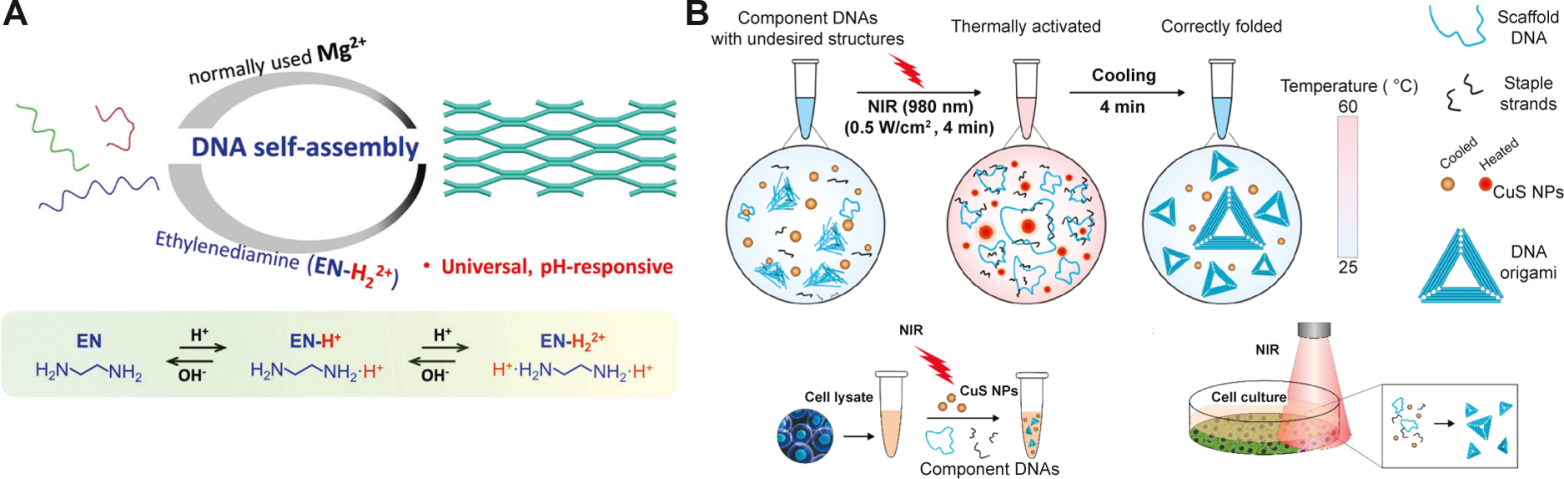
(A) Ethylenediamine mediated folding of DO (Adapted with permission from ref (73), Copyright 2015 John Wiley & Sons, Inc.). (B) Schematic illustration depicting photothermal mediated folding of DO both in cell lysate and cell culture conditions (Adapted with permission from ref (81), Copyright 2021 American Chemical Society).

In addition to the DO design, various nonconventional folding conditions and strategies have been explored in the context of the stability of DO-based assemblies. While cations are typically needed for thermal annealing assisted folding of DO structures,^52,71^ invariably high salt content can be detrimental for both biological and nonbiological applications. In this regard, efforts have been focused on folding DO in salt-free or low salt environments. For example, DNA assemblies, including origami structures, were successfully folded in the presence of polyamine-based compounds like spermidine (Spd^3+^) and ethylenediamine (Figure 2A).^72,73^ This strategy was based on the fact that DNA condensation *in vivo* is promoted by linear polyamine compounds that are fully protonated at physiological pH and can interact with negatively charged DNA.^74,75^ Moreover, linear polyamines are known to stabilize DNA duplex and prevent denaturation from heat.^76^ Furthermore, Simmel and co-workers demonstrated the use of denaturing additives like formamide to fold DO structures in isothermal conditions.^77^ Formamide is known to have a concentration dependent effect on the melting temperature of DNA. The motivation behind this work was to demonstrate DO folding at low temperatures. The methodology consisted of gradually lowering the concentration of formamide from high to low using dialysis, resulting in the formation of DOs at temperatures lower than the usual thermal annealing procedures. Similarly, a nontoxic additive betaine was used to fold origami at room temperature conditions.^78^ Furthermore, in recent work by Rossi-Gendron et al. isothermal folding of DO structures was demonstrated at room temperature using a generic magnesium-free buffer containing NaCl.^79^ These studies may pave the way to incorporating temperature sensitive components like proteins into DO structures during assembly. In an interesting study by Gállego et al.^80^ DO constructs were folded in anhydrous conditions. They utilized a solvent combination of glycerol and choline without divalent ions to demonstrate the formation of both 2D and 3D DO structures. Results of this study might enable the development of nonaqueous approaches for DO assembly for integrating DO with existing top-down nanofabrication procedures, which mostly rely on nonaqueous solvents. In a recent study, Wang and co-workers demonstrated the assembly of DO in cellular environments.^81^ Their approach involved the use of photothermal agents to rapidly heat the buffer solution containing staple strands and scaffold DNA (Figure 2B). Cooling of the solution resulted in the formation of DO, and the entire procedure was completed within 10 min. Moreover, this approach allowed assembling DO in cell culture and cell lysate environments. This work may facilitate the development of remote triggered drug delivery systems where the drug delivery carriers can be rapidly assembled and disassembled.

Understanding various factors influencing the stability and integrity of DO-based assemblies plays important role in the correct evaluation of DO by nanoscopy and microscopy techniques. Atomic force microscopy (AFM) is usually utilized for the so-called single layer structures, whereas transmission electron microscopy (TEM) is often used for multilayer assemblies. AFM imaging in liquid provides high resolution and enables observation of dynamic processes on DO structures; however, DO constructs can be distorted or even damaged by the AFM tip.^28^ AFM imaging in the air is, in a certain sense, more straightforward, as AFM tips and surface deposited DOs are generally more stable under such conditions. However, the resolution in the air is lower compared to imaging in liquid, and surface-deposited DO samples might require careful preparation to remove salt residues and to reduce the deleterious effects originating from liquid to air transfer. For AFM imaging, both in liquid and in air, it is important to remember that the spatial configuration of surface deposited DO-based assemblies might differ from the configuration in solution.^82^ DO–surface interactions are also important in negatively stained TEM (NSTEM) characterization. For example, single layer DOs are flat when deposited on mica for AFM, but they tend to curl when deposited on a carbon grid for TEM,^83^ although the structures can be flattened by the addition of DMSO during TEM sample preparation.^83^ The observed structural configuration of surface-deposited assemblies based on multilayer DO templates might also differ from configurations present in the solution.^84^ This is frequently the case for DO-based plasmonic assemblies.^85−90^ Recently, cryo-electron microscopy (cryo-EM) has emerged as an alternative to NSTEM owing to its ability to characterize DO under native hydrated conditions.^84,91^ Importantly, cryo-EM has opened an avenue for utilizing DO to study other biomolecules, specifically proteins. Here, the efforts have been focused on the design of DO constructs to enable protein structure determination.^92,93^ In addition to design, stability and structural integrity of DO templates will be crucial factors for reaching a near-atomic resolution.

### Strategies for Stabilizing DNA Origami Structures in Solution

#### Ligation and Cross-Linking

Since DO constructs are generally assembled with multiple short ssDNA, they include numerous single strand breaks called nicks, that can compromise the stability and integrity of DO. The most straightforward route for improving the stability of DO structures is through joining staple DNA strands at the nicks. Alternatively, various cross-linking approaches have been investigated for improving the stability and integrity of DO under various conditions.

The process of ligation proceeds by sealing the discontinuities present in the phosphate backbone of DNA, and such a process usually relies on an enzyme called T4 DNA ligase that catalyzes the formation of a phosphodiester bond between 5′-phosphate of one DNA strand and the hydroxyl group of the other DNA strand. Commercially available DNA strands are typically devoid of phosphate groups; therefore, it is necessary to phosphorylate the DO staples with a DNA kinase before ligation. For ligation, DO structures after thermal annealing are typically incubated with T4 ligase at ∼37 °C, as at this temperature the ligation is the most effective. However, the overall ligation efficiency also depends on the extent of staple strand phosphorylation and the DO geometrical design parameters, which determine the accessibility of nicks for ligation. After ligation, the DO constructs exhibit enhanced mechanical and thermal stability and increased resistance to degradation by nucleases when compared to native nonligated structures.^94−97^

While enzymatic ligation can be difficult to implement in higher order origami structures (multilayer structures), the use of chemical methods can negate the restrictions imposed by the geometrical design. In this regard, 1-ethyl-3-(3-(dimethylamino)propyl)carbodiimide hydrochloride (EDC)-catalyzed chemical ligation that forms a phosphate-amine bond between adjacent 5′-phosphorylated and 3′-amine-modified strands can covalently seal the nicks of the assembled DNA nanostructures.^98^ In addition, photoactive molecules in the UV region like 8-methoxypsoralene (8-MOP) and 5-carboxyvinyl-2′-deoxyuridine (CUV) have been used to cross-link DNA nanostructures, thereby enhancing thermal stability.^99−101^ In the case of CUV, the reaction can be reversed by irradiating with 312 nm light. Recently, Dietz and co-workers proposed an elegant strategy for photo-cross-linking of DO structures without the use of additional chemical modifications.^102^ In their approach, they strategically placed thymidines at specific locations enabling thymine dimerization when irradiated at 310 nm. The cross-linked structures were stable at temperatures up to 90 °C in pure double-distilled water, and exhibited enhanced resistivity to degradation by nucleases. Such a light-based cross-linking strategy is particularly attractive as it is simple and does not require external cross-linking agents.^102^ Aside from the cross-linking approaches, the stability of DO structures can also be enhanced by utilizing chemically modified DNA staple strands. Although such chemical approaches were mainly demonstrated for stabilizing scaffold free DNA assemblies, they can be readily extended to DO structures. In this regard, Manetto and co-workers utilized a copper catalyzed click chemistry approach to enhance the stability of DNA constructs. The strategy was based on using alkyne and azide functionalized DNA strands to chemically cross-link the structures via triazole formation, resulting in an enhancement of both temperature stability and resistance to exonuclease damage.^103^ In an interesting study by the Sleiman group, DNA strands modified with hexaethylene glycol and hexanediol chemical moieties as end groups were used to construct DNA nanostructures with improved stability.^104,105^ Such structures had an enhanced lifetime (up to 62 h) in serum compared to native structures. In addition, structures assembled using DNA strands containing unnatural base pairs (2-thiothymidine:A and 5-methyl-isocytidine:isoG) were only partially digested by exonuclease compared to the native structures which were completely degraded.^106^

#### Coating Approaches

**Figure 3:**
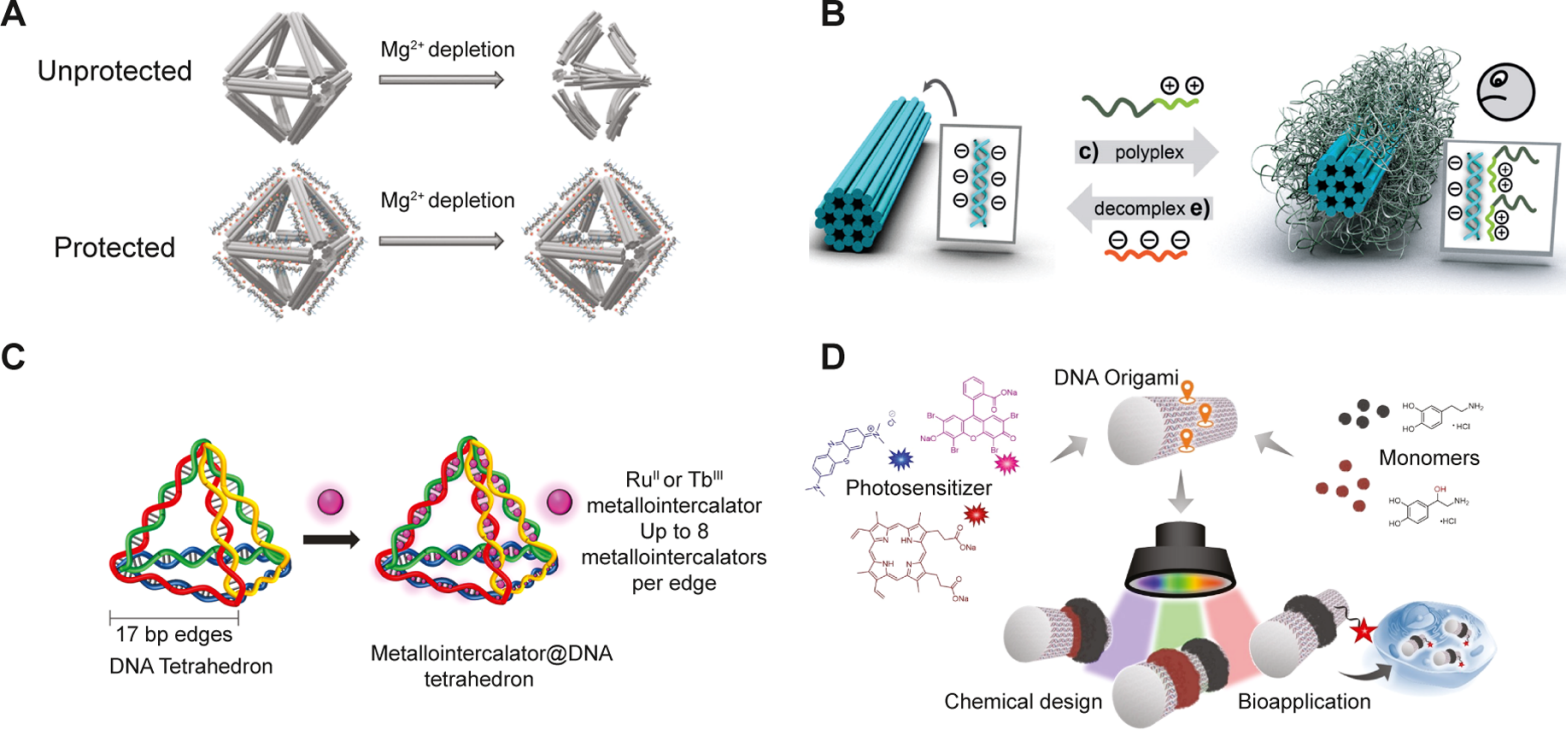
(A) Oligo-peptoid stabilized DO. (Adapted from ref (110), Copyright 2020 The National Academy of Sciences of the USA.) (B) DO coated with positively charged block copolymer micelles. (Adapted with permission from ref (107), Copyright 2017 John Wiley & Sons, Inc.) (C) Supramolecular functionalization of DNS with metallo-intercalator. (Adapted with permission from ref (115), Copyright 2022 American Chemical Society.) (D) Photopolymerization of catecholamine monomers on the surface of DO, in the presence of photosensitizers. (Adapted from ref (121), Copyright 2017 John Wiley & Sons, Inc.)

Non-covalent-based approaches for the enhancement of DO stability typically involve the use of materials that can be immobilized on DO structures through electrostatic interactions. The materials that have been used to coat DO include cationic polymers,^107−109^ peptides,^110−112^ proteins,^113,114^ small molecules,^115,116^ and inorganic precursors^117,118^ (Figure 3A–C). The general strategy of coating is straightforward and does not involve complex chemical functionalization procedures. By appropriately choosing the ratio of positive charges of precursors to negatively charged phosphate groups of DO, successful coating can be achieved without significant aggregation or deformation. Importantly, coatings can confer stability to origami structures in various buffer conditions and protection against nuclease degradation. While coatings enhance the biocompatibility and stability of the DOs, they can also cover the entire surface of origami restricting access to DNA strands for further functionalization. Accessibility of DNA extension in oligolysine-polyethylene glycol (PEG) polymer coated DO by ssDNA strands has been demonstrated recently in collaborative work by the Jungmann and Bastings groups.^119^ Interestingly, the binding kinetics for coated and uncoated DO were indistinguishable. On the other hand, Dietz et al. observed that oligolysine coating reduces the efficiency and utility of blunt end stacking interactions for assembly of higher order DO structures.^112^ Yin and co-workers utilized a dendritic oligonucleotide based coating strategy to protect from nuclease degradation and enhance the stability in low salt conditions.^120^ Furthermore, the oligonucleotide strands can be useful in functionalization with complementary bearing nanoparticles or other entities. In an interesting study by Weil and co-workers, a type of photopolymerization was developed where DO structures were coated with catecholamine based monomeric compounds. Their approach included the use of photosensitizers to initiate the polymerization of catecholamines on the surface of DO in a site-specific manner. When tested under cell culture conditions, polymerized structures provided stability for 24 h compared to native structures (Figure 3D).^121^

In the case of inorganic precursors, efforts have been pursued to utilize DO nanostructures for biomineralization studies. The negatively charged phosphate backbones of DNA structures have a strong affinity to cations and positively charged chemical precursors relevant to biomineralization. Inorganic shells were grown on DNA structures by precisely controlling the coating reaction parameters via the environmental changes^68,122^ (e.g., pH and solvent) and precursor concentrations.^68,117,122−124^ Commonly used precursors include (*N*-[3-(Trimethoxy silyl)propyl]-*N*,*N*,*N*-trimethylammonium chloride (TMAPS), 3-aminopropyl triethoxysilane (APTES), tetraethyl orthosilicate (TEOS), and calcium (Ca^2+^) and magnesium (Mg^2+^) ions. Various studies demonstrated that biomineral shells improve the mechanical properties^122,123,125^ and enhance thermal^122,126^ and biochemical stability^68,127^ of DO-based assemblies. Furthermore, DO technology has provided new opportunities in the engineering of complex inorganic nanostructures with precise shapes and rich types of hybrid materials.^128−130^

**Figure 4:**
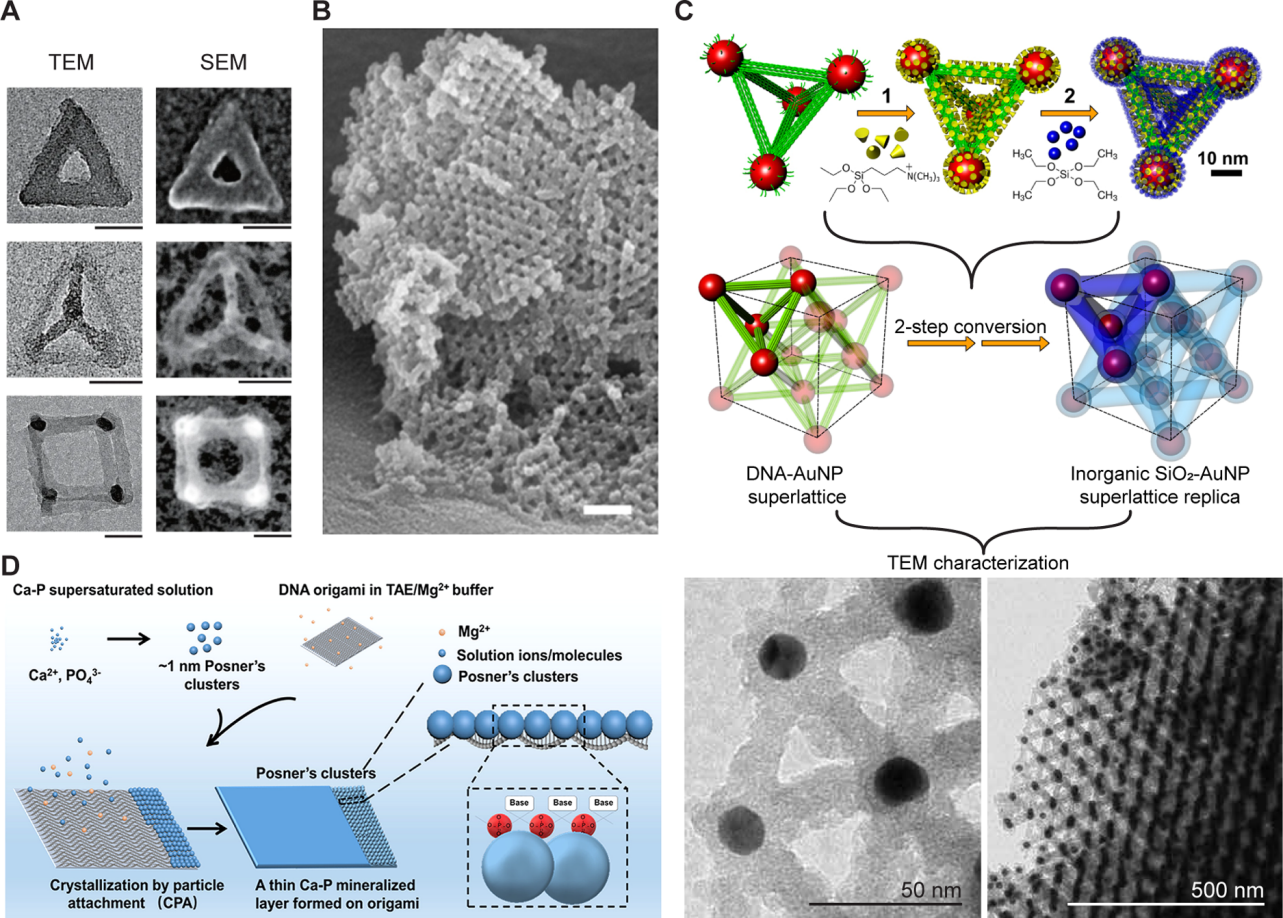
(A) TEM and SEM images of silica coated surface deposited DOs. Scale bars, 50 nm. (Adapted with permission from ref (123), Copyright 2018 Springer Nature.) (B) Silica coated 3D DO crystals. The coating preserves the crystal structure and enables a more detailed structural analysis. Scale bar, 200 nm. (Reproduced with permission from ref (122), Copyright 2019 John Wiley & Sons, Inc.) (C) A superlattice formed by programmable self-assembly of DNA-functionalized Au nanoparticles and silica coating. (Adapted with permission from ref (125), Copyright 2021 American Association for the Advancement of Science.) (D) CaP clusters formed in supersaturated solution and deposited a thin layer on DO-templated biomimetic mineralization. (Adapted with permission from ref (140), Copyright 2020 American Chemical Society.)

Silica is a common inorganic substance that is an important component in hybrid functional materials and the protection of nanostructures.^131−134^ Different approaches have been developed to control the silicification of DO-based assemblies. The interactions between DO and cations such as Mg^2+^ are required to reduce the repulsion between negative charges of phosphate backbones and to maintain their structural integrity. However, the DNA-cation complexations inhibit the formation of hybrid DO-SiO_2_ structures. To overcome this, a cationic coupling agent, e.g., APTES or TMAPS,^68^ is used as an efficient adsorbent on the phosphate groups of origami and as an intermediate layer for the deposition of the silica shell. Liu et al. argued that clusters of the prehydrolyzed mixture of TMAPS and TEOS can promote the silicification process of the substrate deposited 2D and 3D DNA constructs (Figure 4A) and showed that mechanical properties of the origami structures were significantly improved.^123^ A modified Stöber method was utilized by Heuer-Jungemann and colleagues for biomimetic DO silicification with a high origami concentration in a low Mg^2+^ (0.5–3 mM).^122^ Furthermore, the authors showed that silica coating improves the stability of 3D DO crystals (Figure 4B). Recently, our group developed an effective approach for ultrathin silica shell deposition in Mg(OAc)_2_ buffer-free aqueous solution, and controlled silica growth on 3D DO in a mutual solvent of isopropanol and water.^68^ The encapsulated DO@SiO_2_ nanostructures were found to be stable in water and polar organic solvents and were resistant to nuclease-mediated degradation. The combination of programmable self-assembly of DO templates, functionalized Au nanoparticles, together with subsequent silicification enabled the fabrication of various 3D superlattices from face-centered cubic^125^ to tetrahedron^134^ and octahedral^135^ (Figure 4C). Such lattices can exhibit excellent resiliency; e.g., Gang and co-workers showed that silicated DO-based lattices can withstand extreme temperatures (>1000 °C) and pressures (8 GPa).^125^ In addition to coating whole DO structures, protruding double-stranded DNA arrays^136^ and cysteamine^129^ were utilized to achieve site-specific silica growth. Although the mechanism of site-specific silicification is still not fully understood, these studies have highlighted the possibility of fabricating patterned multicomponent composites on DO templates.

Besides silica, calcium phosphate (CaP) coatings have been utilized to biomineralize DO structures. CaP is the most important inorganic component of hard tissues with significant potential for biomaterial applications.^137^ To maintain the structural details and functional moieties from DNA templates, the deposition speed of synthetic CaP on DO has to be precisely controlled. It is known that DNA backbones have a high affinity to Ca^2+^ ions and can therefore induce the nucleation of CaP crystals in a supersaturated solution.^124^ However, the considerable density of negative charge also induces a high local concentration of Ca^2+^ around the DO templates and consequently fast but uncontrollable CaP crystal growth.^124^ Interestingly, CaP nanoclusters (known as Posner’s clusters^138^), which initially form in a supersaturated solution and subsequently adhere to DNA strands, can significantly slow down crystal growth.^139^ In the recent study, Wu and co-workers investigated the calcium mineralization of DO templates in detail (Figure 4D).^140^ Their results indicate that Posner’s clusters deposit on the DNA surface through Mg^2+^/Ca^2+^ exchange due to a higher affinity to phosphate groups. A thin mineral CaP layer was achieved on the desired DNA templates when the reaction was terminated before the massive crystallization. The CaP coated structures exhibited enhanced mechanical and thermal stability when compared to uncoated structures.

#### Strategies for Stabilizing DNA Origami Structures on Surfaces

**Figure 5:**
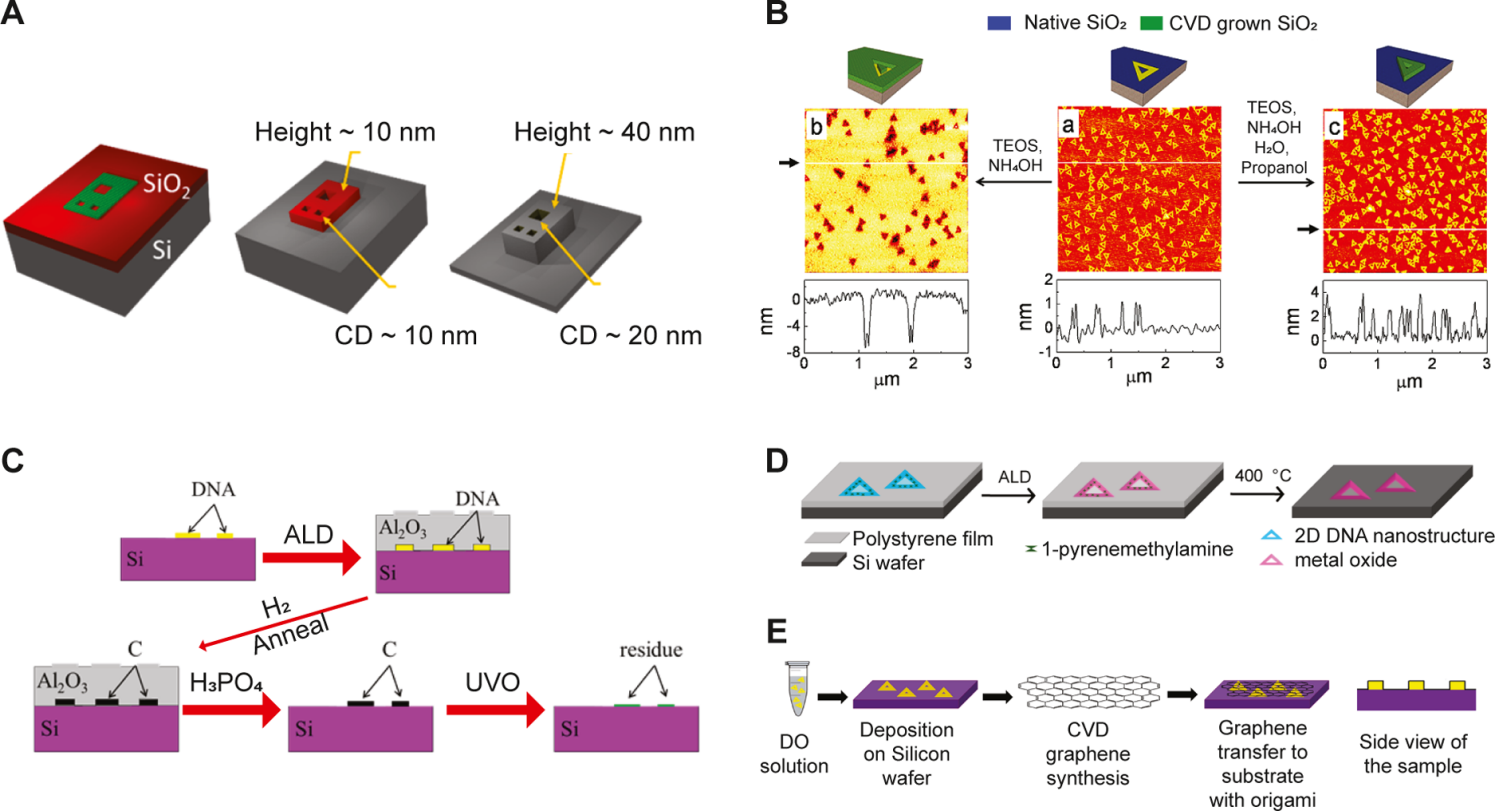
(A) DO as etch masks on Si/SiO_2_ surfaces and respective critical dimension (CD) of features obtained. (Adapted with permission from ref (142), Copyright 2020 American Chemical Society.) (B) Schematic illustration of CVD mediated growth of inorganic oxide materials on DO templates. (Adapted with permission from ref (143), Copyright 2013 American Chemical Society.) (C) Shape conserving carbonization of DO templates by ALD mediated Al_2_O_3_ coating process. (Adapted with permission from ref (147), Copyright 2016 American Chemical Society.) (D) Stabilization of DO on polymer surfaces by 1-pyrenemethylamine. (Adapted with permission from ref (144), Copyright 2020 American Chemical Society.) (E) Graphene encapsulation of DO for high temperature processing. (Adapted with permission from ref (150), Copyright 2018 American Chemical Society.)

While biomineralization approaches enable on-demand fabrication of inorganic nanostructures in solution, the usage of DO has also been extended to surfaces as etch masks for solid surfaces and imprinting masks for polymers surfaces.^43,44,141−145^ To enable functional applications on surfaces, especially for nanofabrication procedures, the focus has been to preserve the morphological features of DO structures. With its remarkably wide array of structures, DO has been at the forefront of producing high resolution templates (∼5 nm) that can potentially offset the challenges faced by the current state-of-the-art lithography techniques. A major challenge has been achieving controlled deposition of DOs on the silicon substrates without compromising their topographical integrity. Since the demonstration of the use of DO as an etch mask to transfer the pattern onto a silicon substrate,^146^ efforts have been put toward developing suitable chemical strategies for stabilizing DOs and preserving their nanoscale features (Figure 5A). In this regard, Surwade et al.^143^ utilized a room temperature-based CVD deposition method to coat origami structures with titanium and silicon oxide layers (Figure 5B). Such a process not only resulted in preserving the origami structure but also allowed the successful transfer of DO defined patterns to the silicon substrate. The process, however, resulted in rough surfaces, probably due to the amorphous nature of material grown by CVD. Using a similar oxide-based strategy, Liu and co-workers produced conductive carbon nanostructures with DO defined morphology. In this process, a 20 nm aluminum oxide (Al_2_O_3_) was deposited using atomic layer deposition (ALD) onto origami structures (Figure 5C).^147^ Subsequently, the structures were processed in a high temperature environment (800–1000 °C) to obtain carbon structures with well-preserved shape and a high degree of crystallinity. However, owing to the hydrophilic nature of both origami and SiO_2_ surfaces, the ALD process resulted in the deposition of oxide layer on both origami and the silicon oxide substrate. To enhance the specificity of oxide coating, Hui et al.^144^ utilized a hydrophobic polymer, i.e., polystyrene, to prevent undesirable background nucleation of oxide material. Furthermore, to facilitate the adsorption and stabilization of origami on hydrophobic surfaces, an amphiphilic molecule 1-pyrene methylamine chloride (PMA) was utilized (Figure 5D). The aromatic basal plane of PMA interacts well with the polystyrene surface, while its amine group can interact with negatively charged DO. Such a strategy resulted in the production of high vertical contrast patterns on Si wafer with antireflective properties.^144^ Although oxide coatings can prevent drying induced collapse of 3D origami structures on Si substrates, such coatings can potentially compromise the vertical contrast and interfere with the RIE process. Recently, Yin and co-workers employed the use of nickel ion (Ni^2+^) assisted stabilization of origami structures, circumventing the need for oxide coating.^148^ The Ni^2+^ ions stabilized the DNA helices by chelating with adjacent DNA helices besides enhancing the adsorption onto the Si substrate. Such a strategy resulted in excellent reproduction of origami features on silicon surfaces with high fidelity. Other strategies preserving topographical features of DO include metal deposition on origami^149^ and encapsulation of origami in graphene^150,151^ to enable high temperature processing conditions (Figure 5E).

#### Self-Healing Strategies

**Figure 6:**
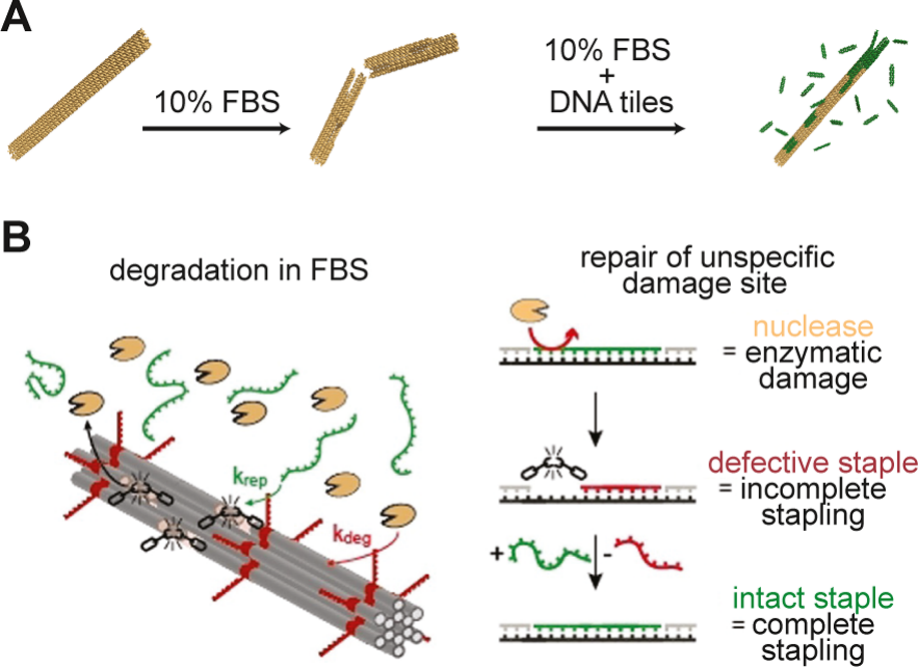
(A) Schematic illustration highlighting DNA tile mediated self-healing of DNA tubes in 10% FBS. (Adapted with permission from ref (152), Copyright 2019 American Chemical Society.) (B) DNA strand mediated self-healing of DO structures in FBS medium. (Adapted with permission from ref (153), Copyright 2017 John Wiley & Sons, Inc.)

Recently, self-healing has been explored as a novel route for improving the stability and integrity of DO-based assemblies. Schulman and co-workers adapted a self-healing-based strategy to stabilize PEG coated DNA tubes in serum (Figure 6A).^152^ Their approach utilized PEG-coated DNA tiles as monomeric species that can accommodate themselves into sites that were damaged due to nuclease induced digestion. Such a process increased the lifetime of PEG-coated DNA tubes to several days in serum, compared to native ones which degraded in 12 h. Using a similar self-healing-based approach, Scheckenbach et al.^153^ demonstrated the use of excess staple strands to facilitate the healing of DO structures in serum conditions (Figure 6B). The study demonstrated successful repair of staples within DO; however, the question of scaffold repair remains open. Such self-healing-based approaches provide an attractive way of repairing DO-based structures under application conditions and can, in principle, be used in tandem with the existing enzymatic ligation methods or cross-linking approaches. Furthermore, a combination of both folding and self-healing in application relevant scenarios could enable the realization of complex programmable DO-based nanostructures and materials with adaptive functional features and life-like properties.

## Conclusions and Future Directions

DO technology has provided material scientists with an advanced set of tools to build materials with unique functionalities. Although various approaches developed for enhancing the stability of DO structures have significantly improved their compatibility with application relevant conditions, challenges remain. For applications of DO-based assemblies in solution, enhancing stability by design (e.g., wireframe design,^62−66^ DNA crossover density^71^) improves structural integrity in a wide range of temperatures, pH values, and cation concentrations and increases resistance to degradation by nucleases. The design-based strategies do not require additional steps after DO folding; however, intensive structure-specific optimization of DNA routing and folding conditions might be necessary.

Cross-linking is an efficient and straightforward way of enhancing the stability of DO structures. However, chemical ligation requires the introduction of additional steps in the DO fabrication process, or chemical modification of DNA stands is needed. As for the light-based cross-linking approaches, they typically rely on UV irradiation, which might induce unwanted damage to other functional components (fluorescent dyes, proteins, etc.) assembled by DO.

Coating provides, perhaps, the most generic and simple route to stabilize DO structures in solution. Polymer-based coating is particularly useful for enhancing DO stability in the context of biomedical applications, whereas coating with inorganic materials, e.g., silica, might enable the fabrication of materials with novel photonic, electronic, and mechanical functionalities. Nevertheless, the effects of coating on the structural integrity of DO-based structures and accessibility of binding sites on DO after coating still require more detailed investigation. Here, computational approaches might offer valuable insight.^65,154^

For applications on surfaces, surface deposition itself often increases the stability of DO-based assembled structures against fluctuations of temperature and pH value.^68^ Here, the main challenge has been the controlled deposition of DO over large areas. Recently, Shetty et al.^155^ demonstrated a low cost for arranging DO over large areas (1 cm^2^). The lithography tools used in this work were based on bottom-up self-assembly that is simple and can be easily scalable. Block copolymer-based self-assembly^156^ is another interesting but not greatly explored approach to facilitate large area patterning of DO structures.

Despite the current challenges, the rapid development of DNA nanotechnology combined with a steadily growing toolbox for enhancing the stability of DO-based-assemblies will open novel routes for the realization of complex programmable nanostructures and materials which are functional not only in the proof of principle but also in application relevant conditions.
